# The Effects of Visual Discriminability and Rotation Angle on 30-Month-Olds’ Search Performance in Spatial Rotation Tasks

**DOI:** 10.3389/fpsyg.2016.01648

**Published:** 2016-10-20

**Authors:** Mirjam Ebersbach, Christian Nawroth

**Affiliations:** ^1^Institut für Psychologie, Universität Kassel, KasselGermany; ^2^School of Biological and Chemical Sciences, Queen Mary University of London, LondonUK

**Keywords:** object permanence, spatial rotation, spatial search, invisible displacements, children, cognitive development

## Abstract

Tracking objects that are hidden and then moved is a crucial ability related to object permanence, which develops across several stages in early childhood. In spatial rotation tasks, children observe a target object that is hidden in one of two or more containers before the containers are rotated around a fixed axis. Usually, 30-month-olds fail to find the hidden object after it was rotated by 180°. We examined whether visual discriminability of the containers improves 30-month-olds’ success in this task and whether children perform better after 90° than after 180° rotations. Two potential hiding containers with same or different colors were placed on a board that was rotated by 90° or 180° in a within-subjects design. Children (*N* = 29) performed above chance level in all four conditions. Their overall success in finding the object did not improve by differently colored containers. However, different colors prevented children from showing an inhibition bias in 90° rotations, that is, choosing the empty container more often when it was located close to them than when it was farther away: This bias emerged in the same colors condition but not in the different colors condition. Results are discussed in view of particular challenges that might facilitate or deteriorate spatial rotation tasks for young children.

## Introduction

Object permanence refers to the knowledge that objects continue to exist even when they are temporarily out of sight. According to Piaget (1954), object permanence develops across several stages in the first two years of life, starting from not searching at all for an object that was hidden in full view of the child, up to tracking and successfully searching for objects that were invisibly displaced. In invisible displacements, an object is hidden in full view of the child in a container. The container is then moved behind a screen, where the object is – invisibly for the child – removed from the container. Thereafter, the empty container is presented to the child. Piaget assumed that only children at the end of the sensorimotor stage can infer that the object should be found behind the screen. This task requires building a mental representation of the object, tracking its displacement while the object itself remains invisible, and inferring the object’s actual position given information of the empty container.

Subsequent research showed that some aspects of object permanence emerge already in children’s first year of life – in particular if looking time or subject’s gaze were used as measure instead of active searching behavior. Baillargeon (1987), for instance, demonstrated that 3.5-month-olds expected that a solid screen rotating back and forth would be stopped by a box placed behind the screen, which indicates that infants knew that the box persisted even when temporarily out of sight. Even 2-month-olds develop assumptions on the persistence and location of objects that are hidden (e.g., Aguiar and Baillargeon, 1999; Hespos and Baillargeon, 2001), and 4-month-olds are able to predictively track the movement of hidden objects with their gaze and head in simple settings (e.g., von Hofsten et al., 2007). This contrasts with findings that even toddlers perform poorly when they have to actively search for hidden objects (e.g., Berthier et al., 2000), but underlines the assumption of a dissociation between representing and acting on hidden objects (Hespos et al., 2009).

A more advanced aspect of object permanence, involving multiple hiding locations, is assessed by *object displacement* tasks, such as spatial transpositions and spatial rotations. In *spatial transpositions*, a target object is hidden in one of two or more containers that interchange their positions. Presented with three containers, children at 20 months of age restricted their search in fact to the two moved containers but still failed to reliably find the object. They showed instead a strong tendency to search at the initial hiding location. It is not before 42 months of age that children search successfully in this task (Sophian, 1984; Barth and Call, 2006).

Another kind of object displacement tasks is the *spatial rotation* of hidden objects, which has been used in the present study. Here, multiple containers are located lined up on a board, which is rotated after a target object was hidden in one of the containers. Barth and Call (2006; see also Herrmann et al., 2007) tested 30-month-old children with this task using three containers that were rotated by 180° or 360°. The target was hidden beneath the left, right, or middle container. In the latter case, no spatial dislocation of the object took place. Children as a group performed significantly below chance level in the 180° condition as they tended to search at the location where the object was initially hidden. In the 360° condition, they performed only at chance level. However, this condition is not instructive as it remains unclear whether correct choices were based on a correct mental representation of the hidden and rotated object or on the tendency to search at the initial hiding location. Similarly, trials in which the object was placed beneath the middle container are not very diagnostic for children’s ability to track hidden objects as the object remained at its location during the rotations.

At first sight, the poor performance of young children in spatial transposition and rotation tasks is astonishing given that infants in their first months of life already show aspects of object permanence in different test setups (e.g., Baillargeon, 1987). Moreover, even infants are able to mentally rotate objects from an early age on, which involves the imagined movement of these object representations (e.g., Örnkloo and von Hofsten, 2007; Moore and Johnson, 2008, 2011; Quinn and Liben, 2008; Frick and Möhring, 2013; Möhring and Frick, 2013). Several reasons might account for children’s particular problems with spatial transposition and rotation tasks which include moving objects. First, children’s memory for the initial hiding location might be so salient that they fail to update this memory during the subsequent movement of the target object (cf. Beran et al., 2005). Second, children might have difficulties in inhibiting a predominant search strategy, even though they are aware of the actual location of the hidden object. They search, for instance, in adjacent hiding containers rather than skipping a clearly empty container (cf. Call, 2001) and tend to search in the container that is located closest to their hand (e.g., the rightmost in a horizontal alignment if they are right-handed or the one closest to them in a vertical alignment of the containers; Diamond, 1991). Third, children might have problems to visually discriminate hiding and non-hiding containers and therefore confuse them.

To check for the latter explanation, Okamoto-Barth and Call (2008) tested 3-year-olds (mean age: 40 months) and older children with a spatial rotation task in which two containers were rotated by 180° and 360°. The visual discriminability of the containers was enhanced by three manipulations. In Experiment 1, (1) the colors of the hiding containers differed (i.e., blue and red), or (2) containers of the same color were placed on differently colored areas on the rotation board. (3) In Experiment 2, the containers were marked by colored stickers (i.e., a red or blue dot). In Experiment 1, 3-year-olds performed above chance in the 360° condition but on chance level in the 180° condition (see also Barth and Call, 2006, for a better performance after 360° rotations compared to 180° rotations) with one exception: Children found the hidden and rotated object more often than expected by chance if it was presented beneath one of two differently colored containers. In Experiment 2, including a different sample of children, 3-year-olds performed above chance with containers that were marked by differently colored stickers and their performance was better than the performance of 3-year-olds in Experiment 1 in the different colors condition. Thus, color seems to be a cue that enhances spatial search in young children. Unfortunately, Okamoto-Barth and Call provided no neutral control condition with unmarked, identical containers on a monochrome board, which would have allowed testing the main effect of color marking.

Further support that 3-year-olds might profit from the visible discriminability of hiding containers in spatial search was provided by Haun et al. (2006). They used three containers with different shapes and different colors and hid an object in one of them. After 1- and 3-year-old children observed the hiding process, the scene was occluded, the containers were transposed, and the occluder was removed. Being prompted to search, 3-year-olds correctly tended to search in the initial hiding container that had been moved to a new position (i.e., feature-based strategy), while 1-year-olds rather searched on the initial position of the hiding container where now a visually different container was located (i.e., place-based strategy).

The effect of identical versus distinctive hiding containers (i.e., boxes with different colors, additionally marked with different stickers) on search behavior was also examined with 22-month-old children in a between-subjects design (Garrad-Cole et al., 2001). A toy was hidden in one of four hiding boxes that were arranged in a rectangle in a room. After the child was placed in the center between the four boxes and turned around with its eyes covered, the child was encouraged to search for the toy. Children in the condition with distinctive hiding boxes were more successful than in the condition with identical hiding boxes to search in the correct box, which indicates that 22-month-olds use object features in their spatial search. However, this task is not comparable with the spatial rotation task used by Barth and Call (2006) as children’s own perspective changed while the object array remained constant in the room, whereas in Barth and Call’s study, the perspective of the children remained stable but the object array was changed. Findings with adults (Simons and Wang, 1998; Wang and Simons, 1999; Creem-Regehr, 2003), infants (Bai and Bertenthal, 1992), and also with dogs (Miller et al., 2009) suggest that performance in spatial rotation tasks that involve changes of one’s own perspective due to self-movements is better than in spatial rotation tasks in which the object arrays are changed.

Taken together, a direct comparison of the effect of same and differently colored containers on children’s searching performance in spatial rotation tasks, in which the hiding containers are moved, is still lacking. In addition, spatial rotations with angles smaller than 180° were not yet tested with young children. Both manipulations, as well as a small number of potential hiding containers, might facilitate the task. By doing so, the ability to solve spatial rotation tasks might emerge in children younger than 3 years of age.

To test this was the aim of the present study. We investigated whether 30-month-olds would be able to track the rotation of hidden objects in a task involving two potential hiding containers, and whether their performance was affected by the discriminability of the hiding containers (i.e., same versus differently colored cups) and by the rotation angle (i.e., 90° versus 180°). A within-subjects design was used that allowed drawing direct conclusions concerning the effects of the independent variables. We expected that visual discriminability of the containers would enhance children’s performance (Nawroth et al., 2015 for a similar effect in goats), and that they would be more successful in 90° than in 180° rotations (cf. Miller et al., 2009 for a similar effect in dogs). In addition, we tested whether boys outperformed girls in tracking rotated objects as previous studies suggested an advantage of boys in performing spatial tasks. For instance, Levine et al. (1999) showed that boys at the age of 4 years and 6 months performed better in spatial transformation tasks than girls of this age. Moreover, mental rotation skills were identified only in 5-month-old boys but not girls of the same age (Moore and Johnson, 2008), and more generally, males often outperform females in mental rotation tasks (Voyer et al., 1995).

## Materials and Methods

### Participants

Participants were 29 children (age: *M* = 30 months, *SD* = 2, range: 28–32 months; 14 boys, 15 girls), which is a sample size comparable to similar studies (e.g., Barth and Call, 2006). They were recruited in various kindergartens and courses offered for young children and their parents (i.e., music and sports courses) in one medium-sized and one small town in Germany.^1^ All children were tested individually.

### Material

**FIGURE 1:**
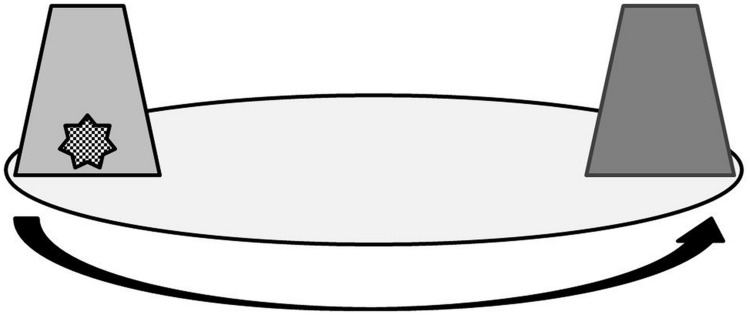
**Schematic setup of the spatial rotation task**.

Two green cups were used as same colored containers, and a green and a yellow cup were used as differently colored containers. The cups were made of opaque plastic with diameter of 7.3 cm and a height of 11.0 cm. In contrast to Barth and Call (2006), the object was hidden beneath one of two (and not of three) cups to reduce the complexity of the task. Five small toys that fitted into the cups were used as target objects. Two of the cups were placed at two opposite edges of a round board (diameter: 36 cm) with a distance of 21.4 cm to each other (**Figure 1**). The board rested on wheels, allowing for being rotated by 90° or 180°. A camera (Panasonic HC-V550) with a tripod was used to record the procedure.

### Design and Procedure

Two factors were manipulated within-subjects: the color of the two cups (i.e., same vs. different colors) and the degree by which the board was rotated (i.e., 90° vs. 180°), resulting in four different conditions. The experiment was split into two sessions, which took place on two different days within two weeks to account for children’s limited ability to concentrate over a long period. In one session, children completed eight trials of the same and eight trials of the different color condition while the board was rotated by 90°. In the other session, eight trials of each color condition were presented with a 180° rotation. In each session, a kindergarten teacher or the mother or father of the child was present to provide emotional comfort to the child because of the child’s young age – but he or she was forbidden to interfere or to communicate during the testing.

At the beginning of each session, the child was allowed to play with the cups and toys to become familiar with the situation for a few minutes. Thereafter, all toys were presented to the child and the first chosen one served as preferred toy that was used throughout all trials of this child. A probe trial without rotation followed to test for simple object permanence. In this trial, the board was located in a distance of about 15 cm in front of the child. Two cups were placed at two opposite edges on the board. The toy was hidden beneath one of the two inverted cups in sight of the child. After being prompted to search for the toy, all children succeeded and progressed to the main test.

In the main test, the child observed the investigator hiding the toy beneath one of the inverted cups and rotating the board by 90° or 180°, depending on the rotation condition, in full sight of the child. As the rotation was finished, the child was immediately prompted to search for the toy. As soon as the child indicated one cup, this decision was recorded and the experimenter lifted the cup.

The orders of color condition (i.e., same versus different first) and rotation condition (i.e., 90° versus 180° first) were counterbalanced between children with one exception: Five children, who were designated for the 180° rotation condition, were absent when this session took place. In addition, one child did not complete the 180° rotation condition with same colors due to a lack of motivation. Furthermore, it was counterbalanced within each child (1) whether the investigator first touched the hiding cup or the non-hiding cup to control for local enhancement, (2) whether the toy was hidden under the left and right cup, (3) whether it was hidden under the green or yellow cup in the condition with differently colored cups, and (4) whether the board with the cups was turned to the left or right. If a child was not successful in the 90° rotation condition, it was noted whether he or she erroneously searched under the cup that was located next to her or him or under the more distant cup. The search behavior of six randomly chosen children was coded by a second rater, yielding an interrater reliability of 0.96 (Cohen’s κ).

## Results

**FIGURE 2:**
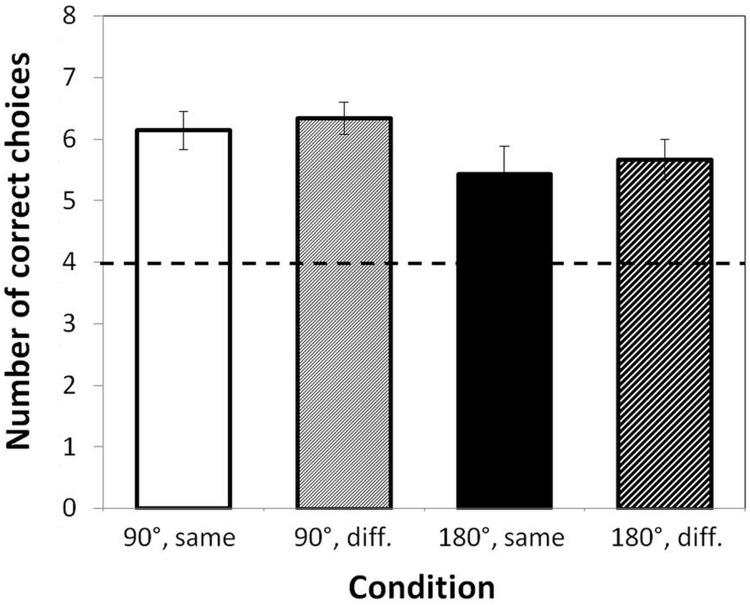
**Mean number of correct choices the four conditions (max.: 8).** Dashed line: chance level. Same, same colored cups; diff., different colored cups. Error bars indicate standard errors.

The mean number of successful trials per condition is shown in **Figure 2**. As preliminary tests indicated that neither the order of the color condition nor the order of the rotation condition yielded significant differences in children’s performance, data were accumulated.

As a group, children found the toy significantly more often than expected by chance (i.e., four correct out of eight trials; see **Figure 2**) in all four conditions: same colors, 90°: *t*(28) = 7.01, *p* < 0.001, different colors, 90°: *t*(28) = 8.89, *p* < 0.001, same colors, 180°: *t*(22) = 3.20, *p* = 0.004, different colors, 180°: *t*(23) = 5.00, *p* < 0.001.

Furthermore, children’s individual performance was examined. In each condition, at least seven out of eight trials had to be correct to assume that a child performed above chance level (Binominal test, one-sided, *p* < 0.05). In the 90° rotation condition, this was the case for 52% of the children (*n* = 15) in the same colors condition and 48% of the children (*n* = 14) in the different colors condition. In the 180° condition, there were 31% of the children in the same colors condition and 31% in the different colors condition (*n* = 9, each) who performed above chance. However, performance differences between the four conditions were not significant, *p* = 0.47 (Chi-square test). Correlational analyses (Spearman’s Rho) revealed a substantial overlap of children in the 90° condition who performed above chance level with same colored cups and with differently colored cups, ρ(*n* = 29) = 0.38, *p* = 0.044. A marginally significant correlation also emerged between children’s above chance performance with differently colored cups in 90° and 180° rotations, ρ(*n* = 24) = 0.37, *p* = 0.078. No other correlations were significant.

To analyze whether rotation angle, color manipulation, or sex yielded significant effects on children’s performance, an ANOVA with repeated measures was computed. A main effect of rotation angle indicated that children found the toy significantly more often in the 90° rotation condition (*M* = 6.41, *SD* = 1.20) than in the 180° rotation condition (*M* = 5.59, *SD* = 1.62), *F*(1,21) = 5.29, *p* = 0.032, $\eta_{p}^{2}$ = 0.20. However, there were no main effects of color condition or sex, nor any interactions, *p*s > 0.10. That is, children did not profit from differently colored cups in their overall search behavior and boys performed similarly as girls.

Finally, to check whether the children in the 90° condition showed an inhibition bias (Barth and Call, 2006) by choosing more often the wrong cup if it was located closer to them compared to the farther cup, the proportion of “closer” errors and the total number of errors was computed. A value of 0.5 would imply that there was no systematic bias. In the same colors condition, children chose significantly more often the wrong cup that was located closer to them (*M* = 0.72, *SD* = 0.30), *t*(20) = 3.31, *p* = 0.003. However, this bias did not emerge in the condition with different colors (*M* = 0.62, *SD* = 0.39), *p* = 0.17.

## Discussion

This study investigated for the first time the effect of visual discriminability of hiding containers in a spatial rotation task including 90° and 180° rotations with 30-month-olds. Children found the hidden object more often than expected by chance in all four conditions, that is, in 90° and 180° rotations involving two visual identical or different containers. The finding that children at the age of 2.5 years were already successful in spatial rotation tasks might be assigned to the simplified task, involving only two hiding containers and also 90° rotations, in which the rotation became more obvious as the array of the hiding containers changed from a horizontal to a vertical orientation. Thus, difficulties of children in previous studies in spatial rotation tasks (e.g., Barth and Call, 2006) could not be assigned to their general inability to track spatial rotations in a motor task, but – at least to some degree – to task complexity.

Visual discriminability of the containers did not improve children’s performance, with one important exception: The bias to erroneously search beneath the closer container in the 90° condition was present with identical containers but disappeared if containers with different colors were involved. Furthermore, children performed significantly better in 90° rotations than in 180° rotations.

Contrary to our expectations, there was no effect of differently colored containers on children’s general success in the search task. This cannot be assigned to ceiling effects as performance on group level and on individual level was not perfect. It can also not be explained by the assumption that children were in general unable to use color as a cue as even infants were shown to use color information, for instance, if they draw statistical inferences. They looked longer if a number of balls of one color were drawn from a box, whose containment was then presented to the infant and which included balls in two colors than if the box contained only balls of the same color as the drawn balls (Xu and Denison, 2009). One explanation for the lacking main effect of color in the present study might be that the cue of different colors was perhaps too weak to generate discriminability. In fact, Okamoto-Barth and Call (2008) reported that monochrome containers marked with differently colored stickers yielded a better performance of 3-year-olds in a spatial rotation task compared to containers with different colors. However, this is not easy to explain from a logical standpoint: Why should the same feature (i.e., different colors) that is applied to the whole hiding containers (i.e., two containers in different colors) be less effective than the same feature that is applied only to a part of the hiding containers (i.e., stickers of different colors on monochrome containers)? As this condition (differently colored containers vs. differently colored stickers) was varied between subjects, a direct comparison of the performance is critical. Nevertheless, it might be assumed that more local cues, such as stickers, or more visually distinct cues, such as shape, might be more effective stimuli to enhance the visual discriminability of hiding containers. Thus, in future studies, one should manipulate not only the color but also the shape of the containers (cf. Nawroth et al., 2015) to enhance visual discriminability. Wilcox (1999), for instance, showed that infants first rely on shape and size when individuating objects and that color cues are used only later in development. Hermer and Spelke (1994, 1996) found that infants did not use color information (i.e., a colored wall in a room) to find a hidden toy after reorientation (but see Learmonth et al., 2001, for contradicting findings).

Another explanation for the lacking main effect of color in the present study might be that color was not diagnostic *per se* because in the different color condition the object was hidden equally often beneath each of the differently colored containers. Thus, color was only diagnostic in each single trial but not in general, and might therefore not have been used by the children. It would nevertheless be interesting to examine in future studies whether color was used if the task is more complex, that is, when more potential hiding containers are involved.

However, the different colors still yielded a relevant effect in the present study, which rules out an alternative explanation that children did not consider the presentation of two different colors at all. If children were presented with containers of different colors, they had no bias to erroneously search in the container located closer to them in the 90° rotation condition. However, this bias emerged in the same colors condition. Thus, different colors can be used by 30-month-olds to prevent an inhibition bias, even though they do not lead to an overall increase of successful searches in spatial rotation tasks. Given this effect and previous findings that color marking improves children’s performance in spatial search (e.g., Okamoto-Barth and Call, 2008), one might argue that children just establish an association between color and object and do not have to track the rotation at all. However, this alternative explanation would only apply if the object is systematically hidden beneath one container. This was not the case in our study (and other studies), where the hiding was counterbalanced between the two containers. Thus, associating one color (or mark) with the object would have led in the next trial to a wrong decision if the hiding container was changed.

Another finding of the present study was that children’s performance was better in the 90° rotation condition than in the 180° rotation condition. This is in line with the performance of dogs in a spatial rotation task, reported by Miller et al. (2009), and is now shown for the first time for children. Miller et al. (2009) argued that 180° rotations might be more difficult than the 90° rotations as the horizontal arrangement of the hiding containers before and after 180° rotations looks identical, whereas arrangement of the containers changes from horizontal to vertical after the 90° rotation. Thus, spatial change is more salient after 90° rotations compared to 180° rotations. Interestingly, the advantage of 90° rotations compared to 180° rotations emerged in the present study not only in the same colors condition but also in the different colors condition – although differently colored cups should make the spatial change obvious in 180° rotations, too, as the yellow and green cup visibly interchange their position. Still finding an advantage of 90° rotations in the different colors condition suggests that it is not only the salience of the spatial change that might account for this advantage. Instead, children’s lacking inhibition to search at the initial hiding location after 180° rotations (Beran et al., 2005), which does not emerge after 90° rotations, might also contribute to their poorer performance after 180° rotations. The fact that the movements of the board in 180° rotations took slightly longer than in 90° rotations and therewith required a slightly longer updating of the object’s location is no plausible alternative explanation for the better performance in 90° rotations as children in the study of Okamoto-Barth and Call (2008) performed better in 360° rotations compared to 180° rotations. Thus, rotation duration does not seem to be a crucial factor in this case. Given that children in the same colors condition showed an inhibition bias by choosing more often the closer than the farther empty cup, one might think of including in future studies also a 90° rotation condition in which the cups are initially presented vertically (i.e., behind each other from the child’s view) and are than rotated by 90° into a horizontal orientation. This might eliminate the inhibition bias and provide a more complete picture of children’s performance in 90° rotations.

Compared to other studies, children in our study were successful in a spatial rotation task at an earlier age (i.e., with 30 months). Barth and Call (2006) reported that 30-month-olds performed below chance level in rotations of 180° and at chance level in rotations of 360°. There is one main difference between their study and the present one. Barth and Call used three potential hiding containers instead of two, which might have enhanced the difficulty for the children. We aimed at reducing the complexity of the task by using only two containers to investigate spatial rotation in its most simple manner. In fact, children in Barth and Call’s study performed the better in spatial search tasks the less the number of containers that were moved (see Sophian, 1984, for a similar effect in spatial transposition tasks). Thus, tracking more (relevant and irrelevant) containers might be more challenging for young children.

Finally, no sex differences were revealed: Boys performed similarly well as girls in the spatial rotation task. This is in line with findings of Levine et al. (1999), who reported no sex differences in children aged 4 years to 4 years 5 months in a spatial transformation task. Only from the age of 4 years 6 months, boys outperformed girls. Thus, potentially sex differences – in particular in action-related spatial tasks, like finding a hidden object – emerge only gradually across childhood. However, to validate this finding for spatial rotation tasks, a larger sample would be necessary.

To sum up, 30-month-olds are already able to solve spatial rotation tasks if only two hiding locations are involved. Their overall performance was not improved by using containers with different colors, potentially as different colors were not efficient enough to generate visual discriminability. However, different colors reduced inhibition errors in the 90° rotation condition. Future studies might investigate whether younger infants benefit from visually discriminable hiding locations also in their overall performance in spatial rotation tasks and whether the degree of rotation affects children’s performance in a consistent manner: Success in tracking hidden objects appears to depend on the cumulative distance of their movements (Franconeri et al., 2010). The finding of a poorer performance in 180° rotations compared to 90° rotations might, at least partly, be assigned to this effect. To test this, a design in which the degree of rotation is varied in several steps until 180° might be instructive.

## Author Contributions

ME planned and conducted the study, analyzed the data, and wrote large parts of the manuscript. CN generated the idea of this study, supported the statistical analyses, and wrote parts of the manuscript.

## Conflict of Interest Statement

The authors declare that the research was conducted in the absence of any commercial or financial relationships that could be construed as a potential conflict of interest.

## Footnotes

1 This study was not connected to any form of formal education or medical treatment and approval by the ethics committee was not required. Nevertheless, we can guarantee that we considered possible ethical issues in this study. Therefore, the used materials were chosen not to pose any undue risk or harm. Informed consent was given by children’s parents in written form to partake in this study. Even after consent was given, participation was on a voluntary basis and children and parents could end their cooperation anytime without any consequences. Proceedings were recorded and participants or their parents were allowed to examine protocols at any time. This proceeding is in accordance with ethical directives specified by the Deutsche Gesellschaft für Psychologie (DGPs).
